# Effects of Malic Acid and Sucrose on the Fermentation Parameters, CNCPS Nitrogen Fractions, and Bacterial Community of *Moringa oleifera* Leaves Silage

**DOI:** 10.3390/microorganisms9102102

**Published:** 2021-10-06

**Authors:** Hanchen Tian, Yue Wang, Zichong Liu, Zhaoying Hu, Yongqing Guo, Ming Deng, Guangbin Liu, Baoli Sun

**Affiliations:** College of Animal Science, South China Agricultural University, Guangzhou 510642, China; thcscau@163.com (H.T.); shanyue687299@163.com (Y.W.); 18319677129@163.com (Z.L.); zhaoyinghu777@163.com (Z.H.); yongqing@scau.edu.cn (Y.G.); dengming@scau.edu.cn (M.D.); gbliu@scau.edu.cn (G.L.)

**Keywords:** *Moringa oleifera* leaves silage, malic acid, sucrose, CNCPS nitrogen fractions, bacterial community

## Abstract

The present study investigated the effects of malic acid, sucrose, and their mixture on the fermentation parameters, Cornell Net Carbohydrate and Protein System (CNCPS) nitrogen fractions, and bacterial community of *Moringa oleifera* leaves (MOL) silages. The trial was divided into four treatments and labeled as CON (control group) and MLA, SUC, and MIX (respectively denoting the addition of 1% malic acid, 1% sucrose, and 1% malic acid + 1% sucrose to the fresh weight basis). The silage packages were opened on the 2nd, 5th, 10th, 20th, and 40th days of ensiling for subsequent determination. Malic acid and sucrose increased the lactic acid content (*p* < 0.05) and pH value, and the acetic acid contents of MLA and MIX were lower than those in CON (*p* < 0.05). Compared with sucrose, malic acid had a better capacity to preserve nutrients and inhibit proteolysis, and thus exerted better effects on the CNCPS nitrogen fractions. The results of 16S rRNA showed that the dominant phyla were *Firmicutes* and *Proteobacteria* and that the dominant genera were *Lactobacillus* and *Weissella*. With the application of silage additives and the processing of fermentation, there was a remarkable change in the composition and function of the bacterial community. The variation of the fermentation parameters and CNCPS nitrogen fractions in the MOL silages caused by malic acid and sucrose might be attributed to the dynamic and dramatic changes of the bacterial community.

## 1. Introduction

*Moringa oleifera* is a perennial tropical deciduous tree belonging to the *Moringaceae* family. It is native to the Himalayan foothills and widely distributed in Southeast Asia, Africa, Central America, and the Arabian Peninsula [1,2]. All parts of *M. oleifera*, including the bark, gum, leaves, and seeds, have an extensive range of uses and contain a variety of bioactive compounds, such as alkaloids, carotenoids, flavonoids, polyphenols, saponins, and vitamins. *M. oleifera* leaves (MOL) have been developed for foods, pharmaceuticals, and health care products [3]. Moreover, MOLs have been reported to have biological functions, including anti-oxidant, anti-diabetes, anti-tumor, and anti-inflammation properties [4]. Given their high content of protein (up to 30% DM) and low content of antinutritional factors, MOLs are widely used in animal husbandry, including poultry, ruminants, nonruminants, and aquatic animals. Previous studies have also shown that MOLs played important roles in promoting growth, enhancing immunity, increasing milk production, improving meat and egg quality, and resistance to heat stress [5,6,7,8]. With such characteristics, MOLs could alleviate the problems of feed shortage and antibiotics abuse to some extent [9].

Ensiling is a traditional and effective method for long-term forage preservation. With the formation of an anaerobic environment, lactic acid bacteria (LAB) multiply and produce organic acids, thereby reducing the pH of forage rapidly and inhibiting the activities of harmful bacteria [10]. However, with fermentation, a large amount of nutrients are broken down by microorganisms. For MOL, the loss rate of protein can even exceed 50% [9]. Hence, silage additives, such as organic acids, sugars, enzymes, and exogenous LAB, have been used to improve fermentation quality and aerobic stability and prevent the destruction of nutrients [11]. Malic acid is widely used in the food industry as an acidifier and preservative. Teleky et al. (2020) reported that malic acid exerts positive effects on sourdough fermentation and decreases the hardness, pH, and moisture content of bread [12]. Another study showed that malic acid has preservation effects on pomegranate fruits at a low temperature, as manifested in the reduced weight loss rate and increased antioxidant activity of fruits [13]. As a feed additive, malic acid plays significant roles in stimulating rumen fermentation, improving microbial efficiency, and decreasing methane production; in addition, malic acid inhibits proteolysis during the ensiling period [14]. As an important part of the citric acid cycle, malic acid can be directly used as a substrate for silage fermentation to reduce the loss of water-soluble carbohydrates (WSCs) and dry matter (DM) [14]. Sucrose serves as a fermentation substrate and promotes the growth of LAB, thereby solving the problem of some protein feeds being difficult to ferment [15]. The quality of silages is greatly determined by the microbial community; with the succession of the microbial community, the chemical composition and fermentation parameters of the forage change accordingly. Thus, further research on microflora may provide theoretical references for improving silage quality. To our knowledge, the research on the effects of malic acid and sucrose on the microbial succession of MOL silages remains limited.

Proposed by Cornell University in the 1990s, the Cornell Net Carbohydrate and Protein System (CNCPS) reflects the nutrient composition and metabolism of ruminant feeds comprehensively [16]. The CNCPS divides proteins into five fractions: PA, instantaneously soluble protein; PB1, rapidly degradable true protein; PB2, intermediately degradable true protein; PB3, slowly degradable protein; PC, undegradable true protein [17,18]. The CNCPS nitrogen fractions of woody forage, such as mulberry and paper mulberry silages, have been reported [15], but no study has explored them for MOL silages.

Hence, in the present study, we investigated the effects of malic acid, sucrose, and their combination on the fermentation parameters, CNCPS nitrogen fractions, and bacterial community of MOL silages. The results of this study may provide a theoretical basis for the utilization of *M. oleifera* as forage.

## 2. Materials and Methods

### 2.1. Silage Material and Preparation

Fresh MOLs were obtained from a commercial plant base located in Chuxiong City, Yunnan Province (101.54° E, 25.05° N). The cultivated variety in this research was PKm2. The trees were cut down when they reached a height of 3 m. After removing the trunk and branches, the leaves and thin branches were collected and chopped to a size of 1–2 cm. The DM content of the MOLs was 243.62 g/kg fresh weight (FW). The WSC, crude protein (CP), neutral detergent fiber (NDF), and acid detergent fiber (ADF) contents of MOLs were 44.77, 279.32, 283.33, and 192.23 g/kg DM, respectively.

After the preparation, the chopped MOLs were divided into four parts and ensiled as follows: (1) without silage additive (CON); (2) with 1% malic acid (purity ≥99.5%) of fresh matter basis (MLA); (3) with 1% sucrose (purity ≥99.5%) of fresh matter basis (SUC); (4) with a mixture of malic acid and sucrose (MIX). After intensive mixing, approximately 200 g of MOLs was placed into a polyethylene bag (20 cm × 30 cm), which was then compacted and sealed using an automatic vacuum packager. A total of 60 bags (4 treatments × 5 silage times × 3 replicates) were obtained and stored at ambient temperature (25–28 °C). At 2, 5, 10, 20, and 40 days of ensiling, three replicates from each of the four treatments were opened for analyses.

### 2.2. Analyses of the Fermentation Parameter and Chemical Composition

Approximately 2 g of fresh sample was maintained at −80 °C from the analyses of the bacterial community. Then, 10 g of fresh sample was mixed with 90 mL of distilled water and stored in a 4 °C refrigerator for 24 h. After filtration using a sterilized four-layer gauze, the filtrate was collected for the determination of the fermentation parameters. Thereinto, pH was measured via a glass electrode pH meter (FE28-Standard, Mettler Toledo, Switzerland). Lactic acid (LA) and volatile fatty acids (VFAs) were tested according to the methods described by Rumsey et al. [19]. Ammonia nitrogen (AN) was determined using phenol-hypochlorite colorimetry [20].

The rest of the fresh sample was dried at 65 °C for 48 h and grounded into powder for the determination of the chemical composition. Briefly, CP and WSC were tested according to the Association of Official Analytical Chemists (AOAC) [21]; NDF and ADF were tested according to Van Soest et al. [22]. True protein (TP), non-protein nitrogen (NPN), neutral detergent insoluble crude protein (NDICP), and acid detergent insoluble crude protein (ADICP) were measured according to the procedure of Licitra et al. [23]. Soluble crude protein (SCP) was measured on the basis of the method described by Yang et al. [24].

The calculation of the CNCPS nitrogen fractions was performed according to previous researches [14,16,17]. The detailed formulas were as follows: PA (%CP) = NPN (%CP), PB1 (%CP) = SCP (%CP) − NPN (%CP), PB3 (%CP) = NDICP (%CP) − ADICP (%CP), PC (%CP) = ADICP (%CP), PB2 (%CP) = 1 − PA (%CP) − PB1 (%CP) − PB3 (%CP) − PC (%CP).

### 2.3. Analyses of the Bacterial Community

The total genomic DNA was extracted via the DNeasy Power Soil Kit (QIAGEN, Inc., Venlo, The Netherlands), and the purity, concentration, and integrity of the DNA samples were determined. Thereafter, the 16S rRNA V3–V4 regions of genomic DNA was amplified via Pyrobest DNA Polymerase (TaKaRa, DR500A) with the primer pairs of 338F (5′-ACTCCTACGGGAGGCAGCA-3′) and 806R (5′-GACTACHVGGGTATCTAATCC-3′). After amplification, Agencourt AMPure Beads (Beckman Coulter, Indianapolis, IN) was adopted for the purification of the PCR products, and the PicoGreen dsDNA Assay Kit (Invitrogen, Carlsbad, CA, USA) was used for quantification. The equimolar and paired-end sequencing (PE250) was performed on the Illumina Novaseq 6000 platform (Personal Biotechnology Co., Ltd., Shanghai, China).

After high-throughput sequencing, QIIME (V 1.8.0) was adopted for the processing of the sequenced data. After the filtration of chimera and low-quality sequences described by previous reports [25,26], the effective sequences were obtained and clustered into operational taxonomic units (OTUs) via UCLUST with a 97% similarity threshold [27]. A representative sequence from each OTU was selected for further taxonomic classification conducted via the Basic Local Alignment Search Tool (BLAST). Subsequently, an OTU table was generated [28].

The bioinformatics analyses of the bacterial community were mainly performed using the QIIME and R softwares (V 4.0.0). In detail, α-diversity was analyzed via the alpha_diversity.py script in QIIME; β-diversity was analyzed by a vegan package in the R software [29]. The functions of microflora were predicted on the basis of the PICRUSt database.

### 2.4. Statistical Analysis

The test data of the fermentation parameters, chemical compositions, CNCPS nitrogen fractions, and α-diversity indices were analyzed using a two-way ANOVA method in the SPSS 25.0 software. The model for data processing was: Y_ij_ = μ + D_i_ + A_j_ + (D ∗ A) _ij_ + ε_ij_, where, Y_ij_ is the dependent variable, μ is the overall mean, D_i_ is the effect of ensiling days, A_j_ is the effect of different silage additives; (D ∗ A) _ij_ is the interaction effect of ensiling days and silage additives, ε_ij_ is the random residual error. The LSD method was adopted for multiple comparisons, and *p* < 0.05 indicated a statistical significance. A Pearson correlation coefficient was adopted for the correlation analysis, with *p* < 0.05 indicating relevance [30].

## 3. Results

### 3.1. Fermentation Parameters of MOL Silages

The fermentation parameters affected by malic acid, sucrose, and their combination are shown in Table 1. The pH values of the MOLs in the four treatments decreased dramatically at the beginning of fermentation (*p* < 0.05); however, the pH values in CON, MLA, and SUC recovered from the 10th day (*p* < 0.05). Compared with that in CON, the pH values in MLA and MIX were significantly lower during the ensiling period (*p* < 0.05). With fermentation, the contents of LA and AN increased obviously (*p* < 0.05). Specifically, the LA contents of MLA, SUC, and MIX were significantly higher than that in CON (*p* < 0.05), whilst the AN content of MLA and MIX were significantly lower than that in CON (*p* < 0.05). The contents of AA increased in the first 10 days of ensiling (*p* < 0.05). Relative to the content in CON, the AA in MLA and MIX decreased (*p* < 0.05). Meanwhile, the WSC contents of CON and SUC decreased during the whole fermentation stage (*p* < 0.05); in MLA and MIX, the reduction of WSC contents occurred in the first 10 days (*p* < 0.05). Toward the end of fermentation, the WSC content of MLA was significantly higher than that of CON (*p* < 0.05).

### 3.2. Chemical Compositions and Nitrogen Fractions of MOL Silage

The results of the chemical compositions are displayed in Table 2. The DM contents in the four treatments changed significantly with fermentation (*p* < 0.05), but no obvious tendency was obtained. However, the DM content in CON was always the minimum compared to those in the other three treatments (*p* < 0.05). The contents of NDF and ADF showed a downtrend as the fermentation proceeded (*p* < 0.05). For the first 20 days, NDF in SUC was always lower than that in CON (*p* < 0.05), but no significant difference was found amongst the four treatments on the last day of ensiling. For ADF, the content in SUC was consistently the minimum (*p* < 0.05). The contents of CP increased with the processing of fermentation (*p* < 0.05), and no significant difference was observed on the last day (*p* > 0.05).

The nitrogen fractions of MOL showed dramatic changes during fermentation (Table 3 and Table 4). The proportions of TP, NDICP, ADICP (PC), PB2, and PB3 showed a descending tendency (*p* < 0.05). Moreover, the ratios of these fractions in MLA and MIX were significantly higher than that in CON (*p* < 0.05). However, NPN (PA), SCP, and PB1 showed opposite tendencies. With fermentation, the ratio of the three fractions increased gradually (*p* < 0.05) and was obviously lower in MLA and MIX than in CON (*p* < 0.05).

### 3.3. Diversity of Bacterial Community in MOL Silage

In the present study, α-diversity, including the observed species. The Chao1 index, Shannon index, Simpson index, and Pielou evenness were studied. As shown in Table 5, with fermentation, no regular tendency was obtained. However, on the 5th, 10th, 20th, and 40th day of ensiling, all the five indices in MLA were the minimum amongst the four treatments (*p* < 0.05). The observed species and Chao1 index in MIX were also significantly lower than those in CON during the whole ensiling stage (*p* < 0.05). In addition, no noticeable difference was noted between CON and SUC as the ensiling proceeded (*p* > 0.05).

**Figure 1:**
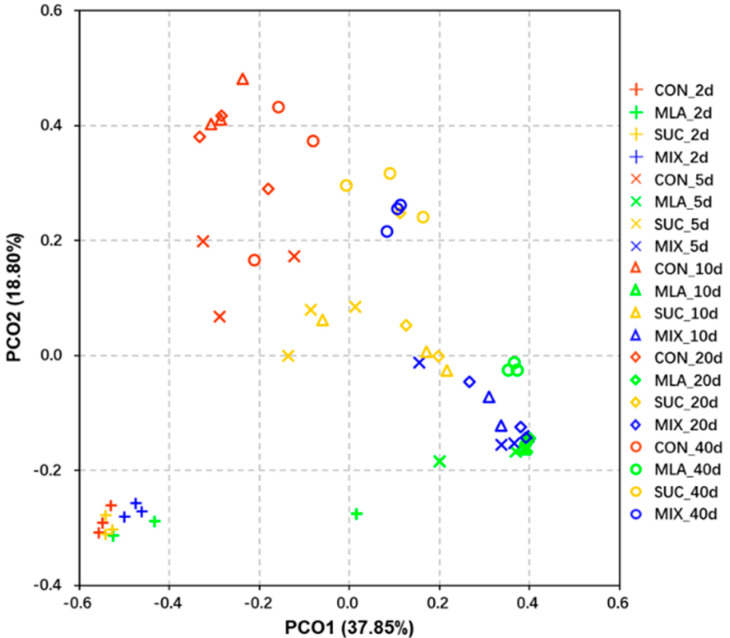
Principal co-ordinates analysis (PCoA) plot of bacterial communities in *Moringa oleifera* leaves silage. CON, control group; MLA, 1% malic acid addition on the fresh weight (FW) basis; SUC, 1% sucrose addition on the FW basis; MIX, 1% malic acid and 1% sucrose addition on the FW basis.

For β-diversity, a principal coordinates analysis (PCoA) was performed, and the results are shown in Figure 1. On the 2nd day of ensiling, no obvious separation was obtained among the four treatments; however, at the 5th, 10th, 20th, and 40th day of fermentation, a remarkable separation was moted between CON and the other three treatments. In addition, significant differences were observed between MLA and SUC, whilst the differences between MLA and MIX on the 5th, 10th, and 20th day were not particularly obvious.

### 3.4. Composition of the Bacterial Community in MOL Silage

**Figure 2:**
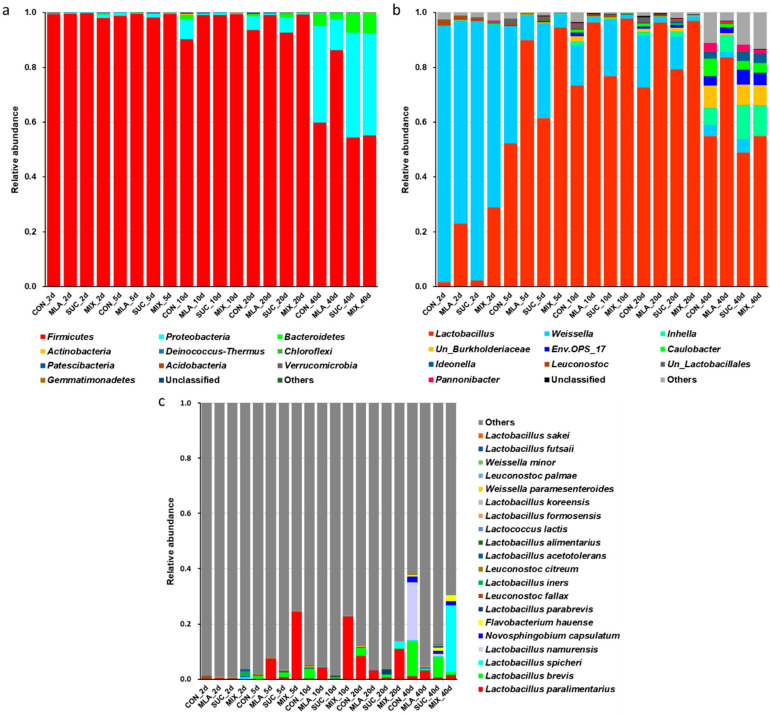
Effect of malic acid and sucrose on the bacterial communities at the phyla level (**a**), genera level (**b**), and species level (**c**) in *Moringa oleifera* leaves silage. CON, control group; MLA, 1% malic acid addition on the fresh weight (FW) basis; SUC, 1% sucrose addition on the FW basis; MIX, 1% malic acid and 1% sucrose addition on the FW basis.

During the 40-day ensiling period, *Firmicutes* was always the most dominant phylum, accounting for more than 90% of the bacterial community on the 5th, 10th, and 20th day (Figure 2a). As the fermentation proceeded, other phyla increased and included *Proteobacteria* and *Bacteroidetes*, especially in CON and SUC. On the 40th day, the relative abundance of *Proteobacteria* and *Bacteroidetes* in CON and SUC was higher than that in MLA. At the genera level (Figure 2b), the most dominant phylum on the 2nd day was *Weissella*, replaced by *Lactobacillus* on the 5th, 10th, 20th, and 40th day. On the 2nd, 5th, 10th, and 20th day of ensiling, *Lactobacillus* in CON and SUC was lower than that in MLA and MIX. On the last day, other genera, such as *Inhella*, unclassified *Burkholderiaceae*, *Env.OPS_17*, *Caulobacter*, and *Ideonella*, were obtained; however, compared with that in MLA, the relative abundance of *Lactobacillus* showed a decline. At the species level, the predominant bacteria were *L. paralimentarius*, *L. brevis*, *L. spicheri*, *L. namurensis*, and *Novosphingobium capsulatum* (Figure 2c). The amount of *L. brevis* in MA was particularly low. The relative abundance of *L. paralimentarius* and *L. spicheri* was high in MIX, whilst that of *L. namurensis* was high in CON.

### 3.5. Predicted Functions and Pathways of Bacterial Community in MOL Silages

**Figure 3:**
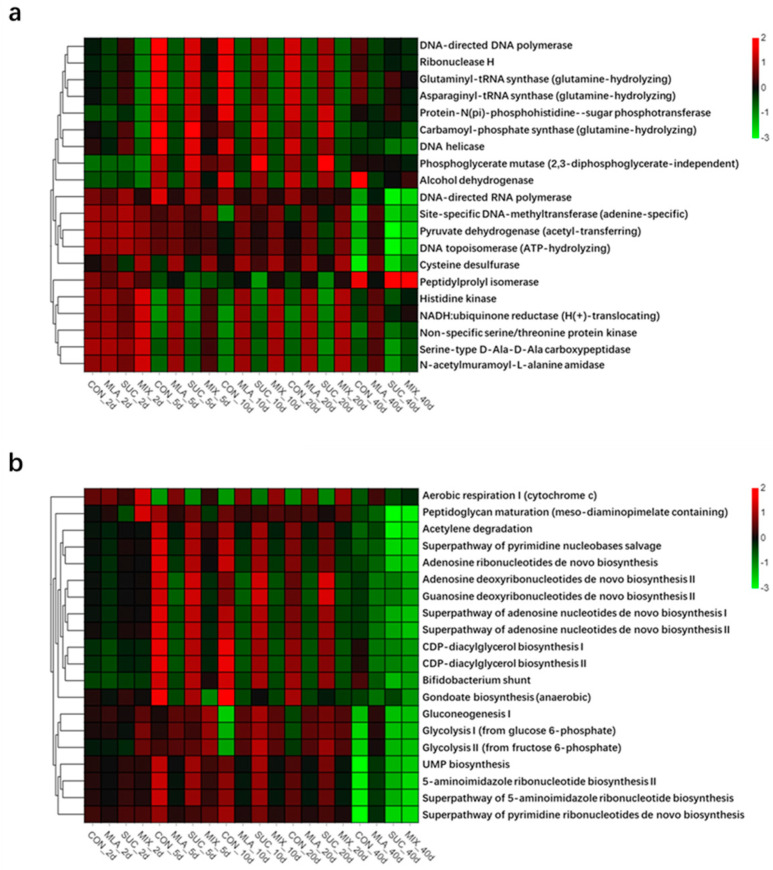
Heatmap of the top 20 predicted functions (**a**) and pathways (**b**) of the bacterial communities analyzed via PICRUSt. CON, control group; MLA, 1% malic acid addition on the fresh weight (FW) basis; SUC, 1% sucrose addition on the FW basis; MIX, 1% malic acid and 1% sucrose addition on the FW basis.

As shown in Figure 3a and Figure S1, the top five predicted functions were DNA helicase, DNA-directed DNA polymerase, Histidine kinase, Non-specific serine/threonine protein kinase, and NADH: ubiquinone reductase (H(+)-translocating). For the predicted pathways (Figure 3b and Figure S2), the top five were Aerobic respiration I (cytochrome c), Acetylene degradation, Superpathway of pyrimidine nucleobases salvage, Peptidoglycan maturation (meso-diaminopimelate containing), and Superpathway of adenosine nucleotides de novo biosynthesis I. The heatmaps of the predicted functions and pathways (Figure 3a,b) implied that the addition of malic acid might have affected the dominant functions and pathways.

**Figure 4:**
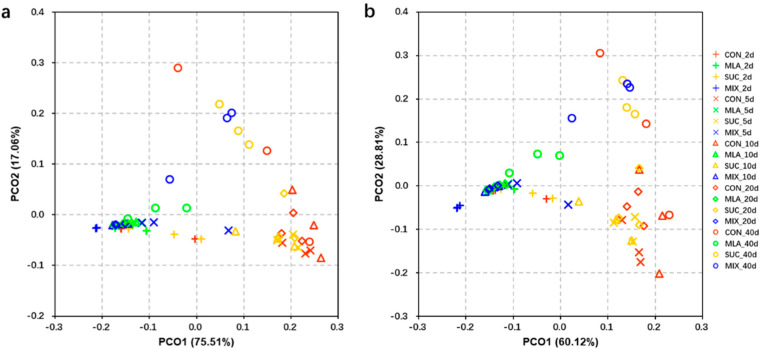
Principal co-ordinates analysis (PCoA) plot of predicted functions (**a**) and pathways (**b**) of the bacterial communities analyzed via PICRUSt. CON, control group; MLA, 1% malic acid addition on the fresh weight (FW) basis; SUC, 1% sucrose addition on the FW basis; MIX, 1% malic acid and 1% sucrose addition on the FW basis.

The PCoA analyses of the predicted functions and pathways were performed. The results showed that the individuals in MLA and MIX always flocked together and that these plots were isolated from the plots in CON and SUC (Figure 4a,b).

### 3.6. Correlation Analysis

**Figure 5:**
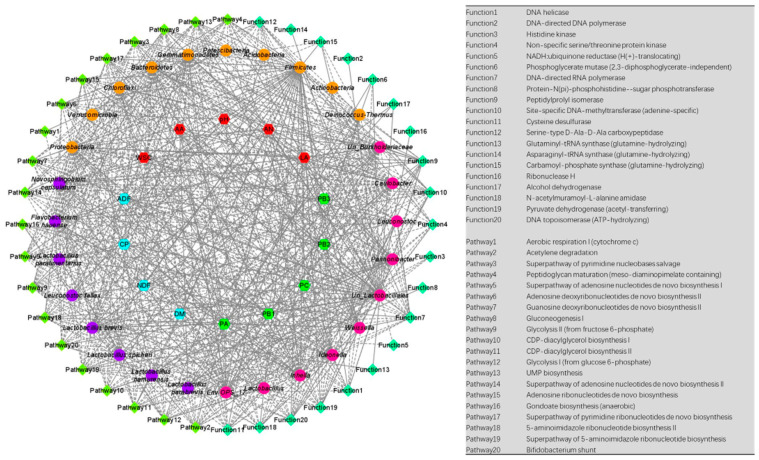
Correlation analyses among various indicators, dominant bacteria, and dominant functions and pathways. The solid line represents a positive correlation, while the dotted line represents a negative correlation.

In the present study, the correlation analysis of various indicators and dominant bacteria was performed (Figure 5 and Table S1). PH, WSC, and ADF were positively correlated with g_*Weissella* (Cor = 0.8670, *p* = 3.39 × 10^−19^; Cor = 0.7922, *p* = 4.72 × 10^−^^14^; Cor = 0.6063, *p* = 2.84 × 10^−7^, respectively). PA had a positive correlation with g_*Env.OPS_17* and p_*Proteobacteria* (Cor = 0.6137, *p* = 1.85 × 10^−7^; Cor = 0.5980, *p* = 4.53 × 10^−7^). PB2 had a positive correlation with p_*Firmicutes* (Cor = 0.6028, *p* = 3.45 × 10^−7^). PH had a negative correlation with g_*Lactobacillus* (Cor = −0.8599, *p* = 1.40 × 10^−18^). LA and CP were negatively correlated with g_*Weissella* (Cor = −0.7310, *p* = 3.32 × 10^−11^; Cor = −0.6106, *p* = 2.21 × 10^−7^, respectively). PB2 had a negative correlation with g_*Env.OPS_17* and p_*Proteobacteria* (Cor = −0.6195, *p* = 1.31 × 10^−7^; Cor = −0.6045, *p* = 3.15 × 10^−7^, respectively). PA had a negative correlation with p_*Firmicutes* (Cor = −0.5958, *p* = 5.12 × 10^−7^).

In addition, we analyzed the correlation between dominant bacteria and dominant functions (Figure 5 and Table S2). In detail, p_*Firmicutes* had a positive correlation with DNA topoisomerase (ATP-hydrolyzing), Pyruvate dehydrogenase (acetyl-transferring), and DNA-directed RNA polymerase (Cor = 0.9325, *p* = 2.50 × 10^−27^; Cor = 0.9065, *p* = 2.22 × 10^−23^; Cor = 0.8841, *p* = 8.09 × 10^−21^, respectively). DNA topoisomerase (ATP-hydrolyzing) had a negative correlation with p_*Proteobacteria* and p_*Bacteroidetes* (Cor = −0.9310, *p* = 4.52 × 10^−27^; Cor = −0.8996, *p* = 1.55 × 10^−22^, respectively). Pyruvate dehydrogenase (acetyl-transferring) was negatively correlated with p_*Proteobacteria* (Cor = −0.9034, *p* = 5.43 × 10^−23^). For the predicted pathways (Figure 5 and Table S3), g_Unclassified_*Lactobacillales* had a positive correlation with Gondoate biosynthesis (anaerobic) (Cor = 0.8509, *p* = 7.36 × 10^−18^). p_*Firmicutes* was positively correlated with Glycolysis I (from glucose 6-phosphate) and Acetylene degradation (Cor = 0.7812, *p* = 1.80 × 10^−13^; Cor = 0.7806, *p* = 1.93 × 10^−13^, respectively). p_*Proteobacteria* was negatively correlated with Acetylene degradation and Glycolysis I (from glucose 6-phosphate) (Cor = −0.7783, *p* = 2.51 × 10^−13^; Cor = −0.7757, *p* = 3.40 × 10^−13^, respectively). g_*Env.OPS_17* had a negative correlation with Peptidoglycan maturation (meso-diaminopimelate containing) (Cor = −0.7725, *p* = 4.85 × 10^−13^).

## 4. Discussion

In this study, the DM content of MOL was 243.62 g/kg FM, which was lower than the ideal content of 30–35% DM reported by Guyader et al. [31]. High moisture increases the risk of spoilage and nutrient loss for silages. Therefore, the use of silage additives is necessary [20]. The CP content of MOL was 279.32 g/kg DM, which was higher than those in the previous studies by 21.5%, 26.0%, and 25.3% [20,32,33]. The differences might be attributed to variations in cultivation, harvest time, planting environment, and climate conditions. WSC is the main fermentation substrate, and its content in this study was 44.77 g/kg DM, which was lower than the minimum requirement of 60–70 g/kg DM for high-quality silages [33]. In sum, MOL is an excellent forage, with high protein and low NDF and ADF. However, the characteristics of high moisture and insufficient WSC hinder the process of ensiling without silage additives.

PH value is an important indicator to evaluate the quality of silages. Generally, when the pH value is less than 4.2, the silage could be regarded as well fermented [34]. In this study, the pH values in MLA and MIX were significantly lower than those in CON and SUC, thereby indicating that malic acid possibly played vital roles. Malic acid could reduce pH rapidly because of its acidity. Moreover, the application of malic acid could promote the growth of LAB, thereby accelerating the production of LA and reducing pH [35]. In addition, sucrose solved the problem of the insufficient fermentation substrate in MOL and promoted LA production. On the 20th day of ensiling, the pH values in CON and SUC presented a dramatic upswing possibly because of the accumulation of AN and other alkaline substances caused by proteolysis [9]. AN reflects the degree of peptide hydrolysis, as well as the deamination of amino acids or peptides [36]. The results of the study indicated that the addition of malic acid reduced the pH value of the silage, thus inhibiting the activity of protease. According to another research, AN production is caused by clostridial fermentation, which might be inhibited by the low pH value created by malic acid [37]. The differences in AA contents in the current work might be associated with the fermentation type. The application of malic acid was speculated to be capable of reducing the abundance of heterofermentative LAB. In addition, propionic acid and butyric acid were undetected during the fermentation period. Butyric acid is the product of secondary fermentation caused by clostridium. It is undesirable and reflects nutritional damage in the forage [32]. This indicates that the preservation of silage is relatively good. Like sucrose, some organic acids (such as citric acid, malic acid, succinic acid, and fumaric acid) and their salts can provide fermentation substrates for silage. This feature might explain why the WSC content in MLA was significantly higher than that in CON during the whole ensiling period [35]. In sum, malic acid and sucrose exerted a positive effect on the fermentation quality of MOL silages, but the addition of the former might be more effective.

The DM of silage was mostly consumed by the metabolism of aerobic microorganisms such as *Clostridia* and yeasts. In general, the lost fractions were mainly digestible organic acids and carbohydrates [38,39]. According to previous studies, malic acid and sucrose could increase the DM content after ensiling [14,15]; however, their combination has not been widely explored. On the last day of ensiling in the current work, the DM contents in MLA and SUC and that in CON showed no significant difference, whilst those in CON and MIX showed obvious differences. This result indicated that relative to malic acid or sucrose alone, their mixture had positive effects on the DM content and reduced DM loss. During the fermentation period, the NDF and ADF contents in SUC were always at a minimum; on the last day, the ADF contents in SUC and MIX were significantly lower than that in CON. This result indicated that sucrose played a crucial role in fiber degradation, which could be attributed to the promotion of acid hydrolysis in plant cells [40]. As in our research, Li et al. reported that the addition of glucose, molasses, sucrose, and their mixtures reduces the NDF and ADF contents of king grass silages [41]; Zhao et al. found that molasses decrease the NDF and ADF contents of rice straw silages [42].

On the last day of ensiling in the current work, no significant difference was found amongst the four treatments, whilst tremendous changes occurred in the protein fractions. The purpose of silages is to extend the storage life of the forage and reduce nutritive losses. Ensiling is a dynamic process accompanied by enzyme and microbial activities [34]. Here, one of the most important biochemical reactions is proteolysis, which transforms proteins into NPN (such as small peptides, free amino acid, and AN) under the activities of microorganisms and proteases [43]. The conversion of TP to NPN (PA fraction) reduces the N utilization in ruminants, further increasing urinary and fecal N losses and causing environmental pollution [44]. Thus, the application of malic acid may have a good effect in terms of nutrient preservation. We can speculate that the addition of malic acid reduces the pH value of silages, thus leading to the decreased activity or even inactivation in protease [35]. SOP consists of PA fraction and PB1 fraction, with PB1 defined as a rapidly degradable true protein, which is lost quickly in the rumen and is difficult to be further utilized. NDICP is composed of a PB3 fraction and a PC fraction (ADICP). NDICP and ADICP belong to proteins bonded with plant cell walls. Relative to NDICP, ADICP cannot be digested and utilized by ruminants [23]. In this study, the proportions of NDICP, PB3, and PC in MLA and MIX were higher than those in CON and SUC. According to our speculation, the enzymes which break chemical bonds that are linked to proteins and structural carbohydrates were inhibited under the acidic conditions caused by malic acid, resulting in the high content of bonding protein [15]. However, because the related research is limited, this topic requires further research. The PB2 fraction is a part of TP for degradation. The increase of PB2 might indicate the promotion of protein digestion in the rumen [45]. Moreover, PB2 + PB3 can be regarded as a ruminal bypass protein or rumen undegradable protein, and its increase means that a large amount of high-quality amino acids can be provided for absorption [46]. In conclusion, malic acid has good properties for avoiding nutrition loss in the forage and could increase the proportion of available proteins. However, it also results in a significant increase in the proportion of undesirable proteins.

Microorganisms play important roles in the ensiling process. Therefore, to make high-quality silages, the dynamic changes of bacterial communities during fermentation should be clearly understood. For the past few years, 16S rRNA high-throughput sequencing technology has been widely used in silage. Changes in community structure are always quantified through a range of nonparametric ecological indices [47]. On the basis of such indices, α-diversity is adopted to measure the richness, diversity, and evenness of species in bacterial communities. In the present study, we analyzed the observed species, the Shannon index, the Simpson index, the Chao1 index, and the Pielou evenness in MOL silages. The observed species, Shannon index, and Simpson index reflect the diversity of the bacterial community. The Chao1 index and Pielou evenness represent the richness and evenness, respectively. The α-diversity indices changed significantly at different ensiling stages, thus indicating that silage is a dynamic microbial reaction. The composition and function of microflora showed great differences as well [48]. The results of this study showed that the addition of malic acid remarkably reduced the five α-diversity indices. Similarly, Zi et al. found that silage king grass silage treated with citric acid had a lower OTU, Chao1 index, ACE index, and Shannon index, possibly because the addition of organic acids inhibits the growth of harmful bacteria [49]. The result of β-diversity reflected the distinction of the bacterial community in each individual or treatment. On the 5th, 10th, 20th, and 40th day of ensiling, CON presented great distinctions from the other three treatments, especially MLA and MIX. In sum, the results of the bacterial diversity corresponded to the fermentation parameters and nitrogen fractions, thus indicating that malic acid might play important roles in regulating bacterial community and thereby affecting silage quality.

The composition of microflora was associated with silage quality. At the phylum level, the dominant bacteria were *Firmicutes* and *Proteobacteria*, similar to those reported by Wang et al. [50]. *Firmicutes* can secrete various cellulases, lipases, and proteases and is able to survive in an anaerobic and low pH environment [51]. In addition, almost all LABs belong to *Firmicutes*. In this study, *Proteobacteria* were positively correlated with AN, thereby indicating that the accumulation of AN increased the pH value and provided favorable conditions for the growth of *Proteobacteria*. A positive correlation was also noted between *Proteobacteria* and PA (NPN), and it suggested the involvement of *Proteobacteria* in proteolysis. On the last day of fermentation, MLA had the lowest abundance of *Proteobacteria*; hence, malic acid might have prevented proteolysis by inhibiting the growth of *Proteobacteria*. The relative abundance of other phyla was inferior, but they also played important roles in the ensiling process. *Bacteroidetes* was positively correlated with PA, LA, and AN and negatively correlated with NDF, ADF, and WSC, probably because *Bacteroidetes* is mainly involved in the hydrolysis of complex macromolecules [52]. In addition, Sa et al. reported that *Chloroflexi* is associated with acid production [53], but our results showed no correlation between *Chloroflexi* and organic acid (LA and AA). We could speculate that *Chloroflexi* only accounted for 0.01% of the bacterial community and that the effects of *Chloroflexi* on fermentation were negligible. For the other phyla, *Acidobacteria*, *Actinobacteria*, *Gemmatimonadetes*, and *Verrucomicrobia* were reported by He et al. [20], whilst *Deinococcus-Thermus* was reported by Dong et al. [54]; however, the roles of these phyla in silages remain unreported. Although we found that these phyla had a correlation with some fermentation parameters and chemical compositions via correlation analyses, the results require further verification and exploration with regard to the mechanism.

At the genera level, the dominant bacteria were *Lactobacillus* and *Weissella*. On the 2nd day of ensiling, *Weissella* accounted for 80% and thus dominated the bacterial community. As fermentation proceeded, the dominance of *Weissella* was gradually replaced by that of *Lactobacillus*. According to a previous study, *Weissella* colonizes at the early stage of ensiling. With the accumulation of organic acid and the decline of the pH value, the growth of *Weissella* is inhibited [55]. On the contrary, a low pH condition favors the growth of *Lactobacillus*. At the initial stage of fermentation, the growth of aerobic bacteria and the respiration of plant cells lead to a high consumption of oxygen. Then, *Lactobacillus* grows rapidly when the environment is completely anaerobic [56]. Notably, the pH value in this work had a positive correlation with *Weissella* and a negative correlation with *Lactobacillus*. The results further indicated that the regulation of malic acid on the two genera might be related to the pH value. *Leuconostoc* is a type of lactate-producing bacteria, and its relative abundance decreased with the processing of ensiling and showed a negative correlation with LA. As a microbial additive generally recognized as safe, *Leuconostoc* is popular in the food fermentation industry and is able to utilize a variety carbon sources, including arabinose, fructose, galactose, glucose, lactose, and sucrose [57]. However, *Leuconostoc* was positively correlated with the pH value and showed a downtrend with the decrease of the pH value. Actually, *Leuconostoc* is regarded as an early colonizer and has poor tolerance to low pH conditions. As fermentation progresses, *Leuconostoc* is gradually replaced by acid-tolerant LABs, such as *Lactobacillus* [58]. As for other genera, *Pannonibacter* and *Inhella* have been reported to exert the effect of biological denitrification and reduce nitrate to nitrite [59,60]; *Caulobacter* plays functional roles in enhancing plant growth by synthesizing siderophores, solubilizing phosphate and producing indole-3-acetic acid [61]. However, the roles of these genera in silage are still undefined and thus require further research.

At the species level, we found that the relative abundance of *L. brevis* was lower in MLA and MIX and that *L. brevis* was positively correlated with AA. As a type of heterofermentative microorganism, *L. brevis* could convert 1 mol glucose to 1 mol LA, 1mol CO_2_, and 1 mol AA [62]. *L. parabrevis* was also found to have a positive relation with AA. Mu et al. reported that the increase of LA and AA in silages was closely related to *L. parabrevis* [63]. In addition, *Le. fallax* was found to proliferate at the heterofermentative stage [64]. Compared with homofermentative fermentation, heterofermentative fermentation is generally characterized by a higher pH, a high AA, and a lower LA content [65]. In the present study, both *L. parabrevis* and *Le. fallax* were inhibited by malic acid, and malic acid regulated the fermentation type by altering the bacterial composition. *L. brevis* showed a positive correlation with AN and PA, but no evidence that *L. brevis* was involved in proteolysis. On the contrary, *L. brevis* has been reported to be a potential silage inoculant capable of mitigating nutrient losses [66]. The proteolysis in CON and MIX might be attributed to the relative high pH caused by heterofermentative fermentation. Some undesirable microorganisms, such as *Clostridium*, were rapidly inhibited when the pH was lower than 4. However, when the pH rose again to 4.5, the activities of the harmful microbes recovered [39]. The rise of pH in CON and SUC at the late stage of ensiling might have accelerated proteolysis to a certain extent. For the other species, *L. spicheri* is considered as a probiotic strain and could be adopted in the production of health care food [67]. *L. paralimentarius* was the most abundant species, but its role in ensiling remains unknown. In the present study, we observed that *L. paralimentarius* was positively correlated with CP and negatively correlated with pH and PB1, thereby suggesting that this microorganism has the potential to be a silage additive.

PICRUSt is a computational approach for predicting the function and pathway compositions of metagenomes according to the databases of reference genomes and marker gene data [68]. In this work, the application of malic acid increased the abundance of Histidine kinase, NADH: ubiquinone reductase (H(+)-translocating), N-acetylmuramoyl-L-alanine amidase, and Serine-type D-Ala-D-Ala carboxypeptidase, etc.; while it decreased the abundance of DNA-directed DNA polymerase, Glutaminyl-tRNA synthase (glutamine-hydrolyzing), DNA helicase, and Alcohol dehydrogenase, etc. In addition, the PCoA analyses of the predicted functions and pathways corroborated the viewpoint that malic acid exerts great effects on the function of bacterial communities. These results could be ascribed to the tremendous variation of some functional bacteria caused by silage additives [39]; however, the mechanisms remain unclear. Moreover, we performed correlation analyses of bacteria and predicted functions, and the results may provide potential biomarkers for the regulation of silage fermentation. In sum, we hypothesized that the regulation of silage quality by malic acid might be related to the composition and function of the microflora.

## 5. Conclusions

In this work, malic and sucrose were able to enhance the fermentation quality of MOL silages. Malic acid regulated the fermentation type by altering the bacterial community. Malic acid also showed a protective effect on the nutrients by inhibiting proteolysis, thereby improving the CNCPS nitrogen composition of MOL silages. Relative to sucrose, malic acid had a remarkable effect on the composition and function of the bacterial community, thereby improving the silage quality. In addition, *L. paralimentarius*, which has not been reported in silages, was found to have the potential to be developed as a silage inoculant. As far as silage preparation is concerned, different additives have different advantages. Therefore, maximizing their respective advantages and forming synergistic effects are the key to the efficient utilization of silages. These steps are worth studying further.

## Figures and Tables

**Table 1:** Fermentation quality of ensiled *Moringa oleifera* leaves treated with malic acid and sucrose.

Items ^1^	Groups ^2^	Days of Silage					SEM ^3^	*p*-Value ^4^		
		2	5	10	20	40		A	D	A × D
pH	CON	4.99 ^Aa^	4.38 ^Ba^	4.22 ^Ca^	4.41 ^Ba^	4.40 ^Ba^	0.04	<0.001	<0.001	<0.001
	MLA	4.83 ^Ab^	4.00 ^BCc^	3.92 ^Cb^	4.03 ^Bc^	4.05 ^Bb^				
	SUC	4.87 ^Ab^	4.23 ^Bb^	4.30 ^BCa^	4.29 ^BCb^	4.35 ^Ca^				
	MIX	4.54 ^Ac^	3.97 ^Bc^	3.95 ^Bb^	3.98 ^Bc^	4.03 ^Bb^				
LA (g/kg DM)	CON	12.35 ^Eb^	18.29 ^Db^	34.84 ^Cc^	60.90 ^Bc^	68.39 ^Ab^	3.35	<0.001	<0.001	<0.001
	MLA	17.16 ^Ea^	25.81 ^Da^	52.53 ^Cb^	73.40 ^Bb^	83.13 ^Aa^				
	SUC	18.32 ^Da^	25.48 ^Ca^	57.93 ^Ba^	79.81 ^Aa^	82.19 ^Aa^				
	MIX	18.72 ^Da^	27.62 ^Ca^	57.84 ^Ba^	79.77 ^Aa^	81.65 ^Aa^				
AA (g/kg DM)	CON	13.82 ^Ca^	14.52 ^Ca^	21.45 ^Aa^	19.63 ^Bb^	21.06 ^Aa^	0.78	<0.001	<0.001	<0.001
	MLA	3.86 ^Bb^	7.97 ^Ab^	8.61 ^Ab^	8.03 ^Ac^	8.75 ^Ab^				
	SUC	14.43 ^Ba^	13.99 ^Ba^	19.50 ^Aa^	22.14 ^Aa^	18.95 ^Aa^				
	MIX	4.51 ^Bb^	4.72 ^Bc^	8.39 ^Ab^	9.28 ^Ac^	8.70 ^Ab^				
WSC (g/kg DM)	CON	26.48 ^Ab^	19.13 ^Bb^	14.89 ^Cb^	11.41 ^Dc^	9.28 ^Eb^	0.98	<0.001	<0.001	0.017
	MLA	34.38 ^Da^	22.53 ^Ca^	17.06 ^Ba^	12.96 ^Aac^	11.13 ^Aa^				
	SUC	29.51 ^Aab^	21.25 ^Bab^	14.77 ^Cb^	12.41 ^CDbc^	10.11 ^Dab^				
	MIX	31.00 ^Aab^	24.28 ^Ba^	14.09 ^Cb^	13.80 ^Ca^	10.47 ^Dab^				
AN (g/kg TN)	CON	66.24 ^Ea^	106.51 ^Da^	95.60 ^Cb^	130.65 ^Ba^	155.23 ^Aa^	5.66	<0.001	<0.001	0.090
	MLA	16.37 ^Cb^	38.05 ^Bb^	41.91 ^Bc^	54.25 ^Bb^	83.37 ^Ab^				
	SUC	58.33 ^Ca^	94.99 ^Ba^	113.84 ^Ba^	140.18 ^Aa^	156.04 ^Aa^				
	MIX	11.10 ^Db^	36.14 ^Cb^	49.05 ^BCc^	57.15 ^Bb^	81.28 ^Ab^				

^1^ LA, lactic acid; AA, acetic acid; WSC, water-soluble carbohydrates; AN, ammonia nitrogen; DM, dry matter; TN, total nitrogen. ^2^ CON, control group; MLA, 1% malic acid addition on the fresh weight (FW) basis; SUC, 1% sucrose addition on the FW basis; MIX, 1% malic acid and 1% sucrose addition on the FW basis. ^3^ SEM, standard error of means. ^4^ A, additives; D, ensiling days; A × D, the interaction effect of additives and ensiling days. Different uppercase indicates significant differences in the same row (*p* < 0.05); different lowercase indicates significant differences in the same column (*p* < 0.05).

**Table 2:** Chemical composition of ensiled *Moringa oleifera* leaves treated with malic acid and sucrose.

Items ^1^	Groups ^2^	Days of Silage					SEM ^3^	*p*-Value ^4^		
		2	5	10	20	40		A	D	A × D
DM (g/kg FW)	CON	224.52 ^Abc^	221.92 ^Ac^	216.75 ^Bb^	226.14 ^Ab^	226.88 ^Ab^	1.01	<0.001	<0.001	0.012
	MLA	237.71 ^Aab^	228.71 ^Bb^	229.23 ^Ba^	233.51 ^ABa^	234.05 ^ABb^				
	SUC	221.77 ^Bc^	229.05 ^ABb^	225.43 ^ABa^	231.82 ^ABa^	234.01 ^Ab^				
	MIX	244.27 ^Aa^	236.61 ^ABa^	228.17 ^BCa^	233.17 ^BCa^	244.89 ^Aa^				
NDF (g/kg DM)	CON	252.90 ^Aa^	242.14 ^Aa^	222.35 ^Ba^	218.08 ^BCa^	205.88 ^Ca^	2.03	<0.001	<0.001	0.275
	MLA	229.62 ^Ab^	227.95 ^ABab^	217.76 ^ABCa^	210.98 ^BCab^	203.34 ^Ca^				
	SUC	211.32 ^Ac^	211.76 ^Ab^	207.12 ^ABa^	204.13 ^ABb^	194.67 ^Ba^				
	MIX	227.37 ^Ab^	221.05 ^Ab^	215.91 ^ABa^	217.02 ^Aab^	199.24 ^Ba^				
ADF (g/kg DM)	CON	179.68 ^Aa^	166.31 ^Ba^	152.21 ^Ca^	150.65 ^Ca^	143.66 ^Ca^	1.49	<0.001	<0.001	0.027
	MLA	165.74 ^Aab^	156.00 ^ABab^	144.79 ^BCa^	145.46 ^BCab^	139.65 ^Cab^				
	SUC	146.56 ^Ac^	145.73 ^Ab^	144.77 ^Aa^	143.70 ^Ab^	137.24 ^Ab^				
	MIX	158.28 ^Abc^	144.75 ^BCb^	146.19 ^Ba^	146.44 ^Bab^	138.22 ^Cb^				
CP (g/kg DM)	CON	273.51 ^Cc^	278.1 1^BCc^	284.83 ^ABb^	284.57 ^ABb^	290.49 ^Aa^	0.96	<0.001	<0.001	0.163
	MLA	278.00 ^Db^	283.98 ^CDb^	287.78 ^BCb^	291.37 ^ABab^	294.25 ^Aa^				
	SUC	287.81 ^BCa^	284.48 ^Cb^	292.38 ^ABab^	295.03 ^Aa^	293.00 ^ABa^				
	MIX	284.86 ^Ba^	294.60 ^Aa^	297.19 ^Aa^	297.54 ^Aa^	295.69 ^Aa^				

^1^ DM, dry matter; FW, fresh weight; NDF, neutral detergent fiber; ADF, acid detergent fiber; CP, crude protein. ^2^ CON, control group; MLA, 1% malic acid addition on the fresh weight (FW) basis; SUC, 1% sucrose addition on the FW basis; MIX, 1% malic acid and 1% sucrose addition on the FW basis. ^3^ SEM, standard error of means. ^4^ A, additives; D, ensiling days; A × D, the interaction effect of additives and ensiling days. Different uppercase indicates significant differences in the same row (*p* < 0.05); different lowercase indicates significant differences in the same column (*p* < 0.05).

**Table 3:** Nitrogen fractions of ensiled *Moringa oleifera* leaves treated with malic acid and sucrose.

Items ^1^	Groups ^2^	Days of Silage					SEM ^3^	*p*-Value ^4^		
		2	5	10	20	40		A	D	A × D
TP (g/kg CP)	CON	755.66 ^Ab^	685.51 ^Bc^	585.91 ^Cb^	473.88 ^Db^	386.88 ^Eb^	16.53	<0.001	<0.001	<0.001
	MLA	813.01 ^Aa^	770.89 ^Ba^	745.91 ^Ba^	655.95 ^Ca^	576.54 ^Da^				
	SUC	724.31 ^Ac^	663.41 ^Bc^	589.16 ^Cb^	427.64 ^Dc^	381.90 ^Eb^				
	MIX	747.76 ^Ab^	725.25 ^Ab^	733.52 ^Aa^	643.34 ^Ba^	592.47 ^Ca^				
NPN (g/kg CP)	CON	244.34 ^Eb^	314.49 ^Da^	414.09 ^Ca^	526.12 ^Bb^	613.12 ^Aa^	16.53	<0.001	<0.001	<0.001
	MLA	186.99 ^Dc^	229.11 ^Cc^	254.09 ^Cb^	344.05 ^Bc^	423.46 ^Ab^				
	SUC	275.69 ^Ea^	336.59 ^Da^	410.84 ^Ca^	572.36 ^Ba^	618.10 ^Aa^				
	MIX	252.24 ^Cb^	274.75 ^Cb^	266.48 ^Cb^	356.66 ^Bc^	407.53 ^Ab^				
SCP (g/kg CP)	CON	299.67 ^Eb^	377.28 ^Da^	479.60 ^Ca^	594.66 ^Bb^	689.65 ^Aa^	17.62	<0.001	<0.001	<0.001
	MLA	228.39 ^Dc^	270.97 ^Cc^	298.73 ^Cb^	392.23 ^Bc^	474.31 ^Ab^				
	SUC	334.20 ^Ea^	392.03 ^Da^	480.55 ^Ca^	640.44 ^Ba^	687.79 ^Aa^				
	MIX	301.30 ^Cb^	321.05 ^Cb^	319.24 ^Cb^	407.73 ^Bc^	461.81 ^Ab^				
NDICP (g/kg CP)	CON	121.23 ^Ac^	109.30 ^Bc^	102.13 ^Cb^	76.29 ^Db^	70.15 ^Ec^	4.26	<0.001	<0.001	<0.001
	MLA	172.86 ^Aa^	158.49 ^Ba^	149.73 ^Ca^	140.49 ^Da^	128.51 ^Ea^				
	SUC	120.23 ^Ac^	92.68 ^Bd^	76.87 ^Cc^	68.27 ^Dc^	65.91 ^Dc^				
	MIX	164.31 ^Ab^	132.04 ^Cb^	141.21 ^Ba^	135.70 ^BCa^	104.95 ^Db^				
ADICP (g/kg CP)	CON	45.96 ^Ab^	45.21 ^Ab^	38.46 ^Bb^	32.23 ^Cb^	30.69 ^Cb^	1.06	<0.001	<0.001	<0.001
	MLA	55.18 ^Aa^	51.96 ^Ba^	50.17 ^Ba^	49.10 ^BCa^	48.12 ^Ca^				
	SUC	45.10 ^Ab^	36.46 ^Bc^	33.30 ^Cc^	30.98 ^Db^	30.96 ^Db^				
	MIX	54.10 ^Aa^	50.15 ^Ba^	49.64 ^Ba^	48.27 ^Ba^	45.99 ^Ca^				

^1^ CP, crude protein; TP, true protein; NPN, non-protein nitrogen; SCP, soluble crude protein; NDICP, neutral detergent insoluble crude protein; ADICP, acid detergent insoluble crude protein. ^2^ CON, control group; MLA, 1% malic acid addition on the fresh weight (FW) basis; SUC, 1% sucrose addition on the FW basis; MIX, 1% malic acid and 1% sucrose addition on the FW basis. ^3^ SEM, standard error of means. ^4^ A, additives; D, ensiling days; A × D, the interaction effect of additives and ensiling days. Different uppercase indicates significant differences in the same row (*p* < 0.05); different lowercase indicates significant differences in the same column (*p* < 0.05).

**Table 4:** CNCPS nitrogen fractions of ensiled *Moringa oleifera* leaves treated with malic acid and sucrose.

Items ^1^	Groups ^2^	Days of Silage					SEM ^3^	*p*-Value ^4^		
		2	5	10	20	40		A	D	A × D
PA (g/kg CP)	CON	244.34 ^Eb^	314.49 ^Da^	414.09 ^Ca^	526.12 ^Bb^	613.12 ^Aa^	16.53	<0.001	<0.001	<0.001
	MLA	186.99 ^Dc^	229.11 ^Cc^	254.09 ^Cb^	344.05 ^Bc^	423.46 ^Ab^				
	SUC	275.69 ^Ea^	336.59 ^Da^	410.84 ^Ca^	572.36 ^Ba^	618.10 ^Aa^				
	MIX	252.24 ^Cb^	274.75 ^Cb^	266.48 ^Cb^	356.66 ^Bc^	407.53 ^Ab^				
PB1 (g/kg CP)	CON	55.33 ^Cab^	62.79 ^BCa^	65.51 ^Ba^	68.54 ^ABa^	76.53 ^Aa^	1.40	<0.001	<0.001	0.246
	MLA	41.40 ^Ac^	41.86 ^Ab^	44.63 ^Ab^	48.18 ^Ab^	50.85 ^Ab^				
	SUC	58.51 ^Ba^	55.45 ^Ba^	69.70 ^Aa^	68.08 ^Aa^	69.69 ^Aa^				
	MIX	49.06 ^ABbc^	46.30 ^Bb^	52.77 ^ABb^	51.07 ^ABb^	54.28 ^Ab^				
PB2 (g/kg CP)	CON	579.10 ^Ab^	513.42 ^Bb^	418.27 ^Cb^	329.05 ^Db^	240.20 ^Eb^	14.08	<0.001	<0.001	<0.001
	MLA	598.75 ^Aa^	570.54 ^ABa^	551.55 ^Ba^	467.29 ^Ca^	397.18 ^Da^				
	SUC	545.56 ^Ac^	515.28 ^Bb^	442.58 ^Cb^	291.29 ^Db^	246.30 ^Eb^				
	MIX	534.40 ^Ac^	546.91 ^Aab^	539.55 ^Aa^	456.57 ^Ba^	433.24 ^Ba^				
PB3 (g/kg CP)	CON	75.28 ^Aa^	64.09 ^Bc^	63.67 ^Bb^	44.06 ^Cb^	39.46 ^Cc^	3.26	<0.001	<0.001	<0.001
	MLA	117.68 ^Aa^	106.53 ^Ba^	99.56 ^BCa^	91.39 ^Ca^	80.39 ^Da^				
	SUC	75.13 ^Ac^	56.22 ^Bc^	43.57 ^Cc^	37.29 ^CDc^	34.96 ^Dc^				
	MIX	110.21 ^Ab^	81.89 ^Cb^	91.57 ^Ba^	87.43 ^BCa^	58.96 ^Db^				
PC (g/kg CP)	CON	45.96 ^Ab^	45.21 ^Ab^	38.46 ^Bb^	32.23 ^Cb^	30.69 ^Cb^	1.06	<0.001	<0.001	<0.001
	MLA	55.18 ^Aa^	51.96 ^Ba^	50.17 ^Ba^	49.10 ^BCa^	48.12 ^Ca^				
	SUC	45.10 ^Ab^	36.46 ^Bc^	33.30 ^Cc^	30.98 ^Db^	30.96 ^Db^				
	MIX	54.10 ^Aa^	50.15 ^Ba^	49.64 ^Ba^	48.27 ^Ba^	45.99 ^Ca^				

^1^ CP, crude protein; PA, instantaneously soluble protein or non-protein nitrogen; PB1, rapid degradable true protein; PB2, intermediately degradable true protein; PB3, slowly degradable protein; PC, undegradable true protein or acid detergent insoluble crude protein. ^2^ CON, control group; MLA, 1% malic acid addition on the fresh weight (FW) basis; SUC, 1% sucrose addition on the FW basis; MIX, 1% malic acid and 1% sucrose addition on the FW basis. ^3^ SEM, standard error of means. ^4^ A, additives; D, ensiling days; A × D, the interaction effect of additives and ensiling days. Different uppercase indicates significant differences in the same row (*p* < 0.05); different lowercase indicates significant differences in the same column (*p* < 0.05).

**Table 5:** Alpha-diversity of bacterial communities of *Moringa oleifera* leaves silage treated with malic acid and sucrose.

Items	Groups ^1^	Days of Silage					SEM ^2^	*p*-Value ^3^		
		2	5	10	20	40		A	D	A × D
Observed species	CON	93.57 ^Ca^	224.70 ^Aa^	141.00 ^BCa^	173.30 ^ABa^	86.20 ^Ca^	7.57	<0.001	<0.001	<0.001
	MLA	75.07 ^Aa^	50.10 ^ABb^	50.53 ^ABb^	44.83 ^Bc^	58.13 ^ABb^				
	SUC	87.53 ^Ba^	206.50 ^Aa^	172.53 ^Aa^	166.90 ^Aa^	91.67 ^Ba^				
	MIX	52.87 ^Ba^	53.93 ^ABb^	56.90 ^ABb^	75.33 ^Ab^	71.07 ^ABb^				
Shannon index	CON	3.13 ^Ca^	6.11 ^Aa^	4.58 ^Ba^	5.29 ^ABa^	4.14 ^BCa^	0.18	<0.001	0.005	<0.001
	MLA	3.39 ^Aa^	2.30 ^Bb^	1.73 ^Bb^	1.55 ^Bc^	2.20 ^Bb^				
	SUC	2.64 ^Ca^	5.68 ^Aa^	4.89 ^ABa^	4.79 ^Ba^	4.50 ^Ba^				
	MIX	3.48 ^ABa^	2.46 ^Cb^	2.72 ^BCb^	2.99 ^BCb^	4.11 ^Aa^				
Simpson index	CON	0.63 ^Bab^	0.95 ^Aa^	0.83 ^Aab^	0.90 ^Aa^	0.86 ^Aa^	0.03	<0.001	0.019	<0.001
	MLA	0.73 ^Aab^	0.54 ^Bb^	0.38 ^BCc^	0.36 ^Cc^	0.50 ^BCb^				
	SUC	0.54 ^Bb^	0.93 ^Aa^	0.86 ^Aa^	0.85 ^Aa^	0.90 ^Aa^				
	MIX	0.80 ^ABa^	0.60 ^Bb^	0.69 ^ABb^	0.64 ^Bb^	0.88 ^Aa^				
Chao1 index	CON	178.40 ^CDa^	419.44 ^Aa^	252.91 ^BCa^	320.24 ^ABa^	143.34 ^Da^	14.94	<0.001	<0.001	<0.001
	MLA	120.03 ^Aa^	82.79 ^Ab^	89.39 ^Ab^	88.24 ^Ac^	107.31 ^Ab^				
	SUC	146.95 ^Ba^	399.11 ^Aa^	316.61 ^Aa^	320.58 ^Aa^	148.33 ^Ba^				
	MIX	57.31 ^Bb^	88.82 ^ABb^	101.87 ^ABb^	131.66 ^Ab^	124.16 ^Aab^				
Pielou evenness	CON	0.48 ^Bab^	0.78 ^Aa^	0.64 ^Aa^	0.71 ^Aa^	0.65 ^Aa^	0.02	<0.001	0.010	<0.001
	MLA	0.55 ^Aab^	0.41 ^Bb^	0.31 ^Bc^	0.28 ^Bc^	0.38 ^Bb^				
	SUC	0.41 ^Cb^	0.74 ^Aa^	0.66 ^ABa^	0.65 ^Ba^	0.69 ^ABa^				
	MIX	0.61 ^ABa^	0.43 ^Cb^	0.47 ^BCb^	0.48 ^BCb^	0.67 ^Aa^				

^1^ CON, control group; MLA, 1% malic acid addition on the fresh weight (FW) basis; SUC, 1% sucrose addition on the FW basis; MIX, 1% malic acid and 1% sucrose addition on the FW basis. ^2^ SEM, standard error of means. ^3^ A, additives; D, ensiling days; A × D, the interaction effect of additives and ensiling days. Different uppercase indicates significant differences in the same row (*p* < 0.05); different lowercase indicates significant differences in the same column (*p* < 0.05).

## Data Availability

The sequences in this study were submitted to the Sequence Read Archive (SRA), and a BioProject number PRJNA748826 was obtained.

## Supplementary Materials

The following are available online at https://www.mdpi.com/article/10.3390/microorganisms9102102/s1, Figure S1: The top 20 predicted functions of the bacterial communities analyzed via PICRUSt. CON, control group; MLA, 1% malic acid addition on the fresh weight (FW) basis; SUC, 1% sucrose addition on the FW basis; MIX, 1% malic acid and 1% sucrose addition on the FW basis; Figure S2: The top 20 predicted pathways of the bacterial communities analyzed via PICRUSt. CON, control group; MLA, 1% malic acid addition on the fresh weight (FW) basis; SUC, 1% sucrose addition on the FW basis; MIX, 1% malic acid and 1% sucrose addition on the FW basis; Table S1: Correlation analysis between various indicators and dominant bacteria (phyla, genera, and species level); Table S2: Correlation analysis between dominant predicted functions and dominant bacteria (phyla, genera, and species); Table S3: Correlation analysis between dominant predicted pathways and dominant bacteria (phyla, genera, and species).

## References

[1] Çelekli A., Al-Nuaimi A.I., Bozkurt H. (2019). Adsorption kinetic and isotherms of Reactive Red 120 on *Moringa oleifera* seed as an eco-friendly process. J. Mol. Struct..

[2] Pagano C., Perioli L., Baiocchi C., Bartoccini A., Beccari T., Blasi F., Calarco P., Ceccarini M.R., Cossignani L., di Michele A. (2020). Preparation and characterization of polymeric microparticles loaded with *Moringa oleifera* leaf extract for exuding wound treatment. Int. J. Pharmaceut..

[3] Castro-López C., Espinoza-González C., Ramos-González R., Boone-Villa V.D., Aguilar-González M.A., Martínez-Ávila G.C.G., Aguilar C.N., Ventura-Sobrevilla J.M. (2021). Spray-drying encapsulation of microwave-assisted extracted polyphenols from *Moringa oleifera*: Influence of tragacanth, locust bean, and carboxymethyl-cellulose formulations. Food. Res. Int..

[4] Tiloke C., Anand K., Gengan R.M., Chuturgoon A.A. (2018). *Moringa oleifera* and their phytonanoparticles: Potential antiproliferative agents against cancer. Biomed. Pharmacother..

[5] Cui Y., Wang J., Lu W., Zhang H., Wu S., Qi G. (2018). Effect of dietary supplementation with *Moringa oleifera* leaf on performance, meat quality, and oxidative stability of meat in broilers. Poult. Sci..

[6] El-Kassas S., Abdo S.E., Abosheashaa W., Mohamed R., Moustafa E.M., Helal M.A., El-Naggar K. (2020). Growth performance, serum lipid profile, intestinal morphometry, and growth and lipid indicator gene expression analysis of mono-sex Nile tilapia fed *Moringa oleifera* leaf powder. Aquacult. Rep..

[7] Kekana T.W., Marume U., Muya M.C., Nherera-Chokuda F.V. (2020). Periparturient antioxidant enzymes, haematological profile and milk production of dairy cows supplemented with *Moringa oleifera* leaf meal. Anim. Feed Sci. Tech..

[8] N’nanle O., Tété-Bénissan A., Nideou D., Onagbesan O.M., Tona K. (2020). Use of *Moringa oleifera* leaves in broiler production chain. 1—Effect on Sasso breeder hens performances, internal quality of hatching eggs and serum lipids. Vet. Med. Sci..

[9] He L., Lv H., Chen N., Wang C., Zhou W., Chen X., Zhang Q. (2020). Improving fermentation, protein preservation and antioxidant activity of *Moringa oleifera* leaves silage with gallic acid and tannin acid. Bioresour. Technol..

[10] Ren H., Feng Y., Pei J., Li J., Wang Z., Fu S., Zheng Y., Li Z., Peng Z. (2020). Effects of *Lactobacillus plantarum* additive and temperature on the ensiling quality and microbial community dynamics of cauliflower leaf silages. Bioresour. Technol..

[11] Wu Z., Luo Y., Bao J., Luo Y., Yu Z. (2020). Additives affect the distribution of metabolic profile, microbial communities and antibiotic resistance genes in high-moisture sweet corn kernel silage. Bioresour. Technol..

[12] Tekely B.-E., Martău G.A., Vodnar D.C. (2020). Physicochemical effects of *Lactobacillus plantarum* and *Lactobacillus casei* cocultures on soy–wheat flour dough fermentation. Foods.

[13] Ehteshami S., Abdollahi F., Ramezanian A., Rahimzadeh M., Dastjerdi A.M. (2020). Maintenance of quality and bioactive compounds of cold stored pomegranate (*Punica granatum* L.) fruit by organic acids treatment. Food Sci. Technol. Int..

[14] Ke W.C., Ding W.R., Ding L.M., Xu D.M., Zhang P., Li F.H., Guo X. (2018). Influences of malic acid isomers and their application levels on fermentation quality and biochemical characteristics of alfalfa silage. Anim. Feed Sci. Tech..

[15] Wang X., Liu H., Xie Y., Zhang Y., Lin Y., Zheng Y., Yang X., Wang N., Ni K., Yang F. (2021). Effect of sucrose and lactic acid bacteria additives on fermentation quality, chemical composition and protein fractions of two typical woody forage silages. Agriculture.

[16] Dineen M., McCarthy B., Ross D., Ortega A., Dillon B., Van Amburgh M.E. (2021). Characterization of the nutritive value of perennial ryegrass (*Lolium perenne* L.) dominated pastures using updated chemical methods with application for the Cornell Net Carbohydrate and Protein System. Anim. Feed Sci. Tech..

[17] Nie H., Wang Z., You J., Zhu G., Wang H., Wang F. (2020). Comparison of in vitro digestibility and chemical composition among four crop straws treated by *Pleurotus ostreatus*. Asian Austral. J. Anim..

[18] Gholizadeh H., Naserian A.A., Yari M., Jonker A., Yu P. (2021). Crude protein fractionation, in situ ruminal degradability and FTIR protein molecular structures of different cultivars within barley, corn and sorghum cereal grains. Anim. Feed Sci. Tech..

[19] Rumsey T.S., Noller C.H., Rhykerd C.L., Burns J.C. (1967). Measurement of Certain Metabolic Organic Acids in Forage, Silage, and Ruminal Fluid by Gas-Liquid Chromatography. J. Dairy Sci..

[20] He L., Lv H., Xing Y., Chen X., Zhang Q. (2020). Intrinsic tannins affect ensiling characteristics and proteolysis of *Neolamarckia cadamba* leaf silage by largely altering bacterial community. Bioresour. Technol..

[21] AOAC (2002). Official Methods of Analysis.

[22] Van Soest P.J., Robertson J.B., Lewis B.A. (1991). Methods for dietary fiber, neutral detergent fiber, and nonstarch polysaccharides in relation to animal nutrition. J. Dairy Sci..

[23] Licitra G., Hernandez T.M., Van Soest P.J. (1996). Standardization of procedures for nitrogen fractionation of ruminant feeds. Anim. Feed Sci. Tech..

[24] Yang L., Christensen D.A., McKinnon J.J., Beattie A.D., Yu P. (2013). Effect of altered carbohydrate traits in hulless barley (*Hordeum vulgare* L.) on nutrient profiles and availability and nitrogen to energy synchronization. J. Cereal Sci..

[25] Gill S.R., Pop M., DeBoy R.T., Eckburg P.B., Turnbaugh P.J., Samuel B.S., Gordon J.I., Relman D.A., Fraser-Liggett C.M., Nelson K.E. (2006). Metagenomic analysis of the human distal gut microbiome. Science.

[26] Chen H., Jiang W. (2014). Application of high-throughput sequencing in understanding human oral microbiome related with health and disease. Front. Microbiol..

[27] Malik I., Batra T., Das S., Kumar V. (2020). Light at night affects gut microbial community and negatively impacts host physiology in diurnal animals: Evidence from captive zebra finches. Microbiol. Res..

[28] Urbanek A.K., Rybak J., Wróbel M., Leluk K., Mirończuk A.M. (2020). A comprehensive assessment of microbiome diversity in *Tenebrio molitor* fed with polystyrene waste. Environ. Pollut..

[29] De Filippis F., Parente E., Zotta T., Ercolini D. (2018). A comparison of bioinformatic approaches for 16S rRNA gene profiling of food bacterial microbiota. Int. J. Food Microbiol..

[30] Hu Z., Yang Y., Lu L., Chen Y., Zhu Z., Huang J. (2021). Kinetics of water absorption expansion of rice during soaking at different temperatures and correlation analysis upon the influential factors. Food Chem..

[31] Guyader J., Baron V.S., Beauchemin K.A. (2018). Corn forage yield and quality for silage in short growing season areas of the Canadian prairies. Agronomy.

[32] Wang Y., He L., Xing Y., Zheng Y., Zhou W., Pian R., Yang F., Chen X., Zhang Q. (2019). Dynamics of bacterial community and fermentation quality during ensiling of wilted and unwilted *Moringa oleifera* leaf silage with or without lactic acid bacterial inoculants. mSphere.

[33] Wang Y., He L., Xing Y., Zhou W., Pian R., Yang F., Chen X., Zhang Q. (2019). Bacterial diversity and fermentation quality of *Moringa oleifera* leaves silage prepared with lactic acid bacteria inoculants and stored at different temperatures. Bioresour. Technol..

[34] Wang M., Wang L., Yu Z. (2019). Fermentation dynamics and bacterial diversity of mixed lucerne and sweet corn stalk silage ensiled at six ratios. Grass Forage Sci..

[35] Guo X.S., Bai J., Li F.H., Xu D.M., Zhang Y.X., Bu D.P., Zhao L.S. (2020). Effects of malate, citrate, succinate and fumarate on fermentation, chemical composition, aerobic stability and digestibility of alfalfa silage. Anim. Feed Sci. Tech..

[36] Bai J., Xu D., Xie D., Wang M., Li Z., Guo X. (2020). Effects of antibacterial peptide-producing *Bacillus subtilis* and *Lactobacillus buchneri* on fermentation, aerobic stability, and microbial community of alfalfa silage. Bioresour. Technol..

[37] Mu L., Xie Z., Hu L., Chen G., Zhang Z. (2020). Cellulase interacts with *Lactobacillus plantarum* to affect chemical composition, bacterial communities, and aerobic stability in mixed silage of high-moisture amaranth and rice straw. Bioresour. Technol..

[38] Yuan X., Wen A., Dong Z., Desta S.T., Shao T. (2017). Effects of formic acid and potassium diformate on the fermentation quality, chemical composition and aerobic stability of alfalfa silage. Grass Forage Sci..

[39] Wang C., Zheng M., Wu S., Zou X., Chen X., Ge L., Zhang Q. (2021). Effects of Gallic Acid on Fermentation Parameters, Protein Fraction, and Bacterial Community of Whole Plant Soybean Silage. Front. Microbiol..

[40] Ni K., Wang F., Zhu B., Yang J., Zhou G., Pan Y., Tao Y., Zhong J. (2017). Effects of lactic acid bacteria and molasses additives on the microbial community and fermentation quality of soybean silage. Bioresour. Technol..

[41] Li M., Zi X., Zhou H., Hou G., Cai Y. (2014). Effects of sucrose, glucose, molasses and cellulase on fermentation quality and in vitro gas production of king grass silage. Anim. Feed Sci. Tech..

[42] Zhao J., Dong Z., Li J., Chen L., Bai Y., Jia Y., Shao T. (2019). Effects of lactic acid bacteria and molasses on fermentation dynamics, structural and nonstructural carbohydrate composition and in vitro ruminal fermentation of rice straw silage. Asian Austral. J. Anim..

[43] Martens S.D., Korn U., Roscher S., Pieper B., Schafft H., Steinhöfel O. (2019). Effect of tannin extracts on protein degradation during ensiling of ryegrass or lucerne. Grass Forage Sci..

[44] Dentinho M.T.P., Paulos K., Portugal P.V., Moreira O.C., Santos-Silva J., Bessa R.J.B. (2019). Proteolysis and in situ ruminal degradation of lucerne ensiled with *Cistus ladanifer* tannins. Grass Forage Sci..

[45] Li X., Tian J., Zhang Q., Jiang Y., Hou Z., Wu Z., Yu Z. (2018). Effects of applying *Lactobacillus plantarum* and Chinese gallnut tannin on the dynamics of protein degradation and proteases activity in alfalfa silage. Grass Forage Sci..

[46] Brand T.S., Jordaan L. (2020). Effect of extrusion on the rumen undegradable protein fraction of lupins. S. Afr. J. Anim. Sci..

[47] Spatharis S., Roelke D.L., Dimitrakopoulos P.G., Kokkoris G.D. (2011). Analyzing the (mis)behavior of Shannon index in eutrophication studies using field and simulated phytoplankton assemblages. Ecol. Indic..

[48] Sepehri A., Sarrafzadeh M.-H. (2019). Activity enhancement of ammonia-oxidizing bacteria and nitrite-oxidizing bacteria in activated sludge process: Metabolite reduction and CO_2_ mitigation intensification process. Appl. Water Sci..

[49] Zi X., Li M., Chen Y., Lv R., Zhou H., Tang J. (2021). Effects of citric acid and *Lactobacillus plantarum* on silage quality and bacterial diversity of king grass silage. Front. Microbiol..

[50] Wang Y., Wang C., Zhou W., Yang F., Chen X., Zhang Q. (2018). Effects of Wilting and *Lactobacillus plantarum* Addition on the Fermentation Quality and Microbial Community of *Moringa oleifera* Leaf Silage. Front. Microbiol..

[51] Yuan X., Dong Z., Liu J., Shao T. (2020). Microbial community dynamics and their contributions to organic acid production during the early stage of the ensiling of Napier grass (*Pennisetum purpureum*). Grass Forage Sci..

[52] Zhao X., Liu J., Liu J., Yang F., Zhu W., Yuan X., Hu Y., Cui Z., Wang X. (2017). Effect of ensiling and silage additives on biogas production and microbial community dynamics during anaerobic digestion of switchgrass. Bioresour. Technol..

[53] Sa D.W., Lu Q., Wang Z., Ge G., Sun L., Jia Y. (2021). The potential and effects of saline-alkali alfalfa microbiota under salt stress on the fermentation quality and microbial. BMC Microbiol..

[54] Dong Z., Shao T., Li J., Yang L., Yuan X. (2020). Effect of alfalfa microbiota on fermentation quality and bacterial community succession in fresh or sterile Napier grass silages. J. Dairy Sci..

[55] Graf K., Ulrich A., Idler C., Klocke M. (2016). Bacterial community dynamics during ensiling of perennial ryegrass at two compaction levels monitored by terminal restriction fragment length polymorphism. J. Appl. Microbiol..

[56] Dunière L., Sindou J., Chaucheyras-Durand F., Chevallier I., Thévenot-Sergentet D. (2013). Silage processing and strategies to prevent persistence of undesirable microorganisms. Anim. Feed Sci. Tech..

[57] Díaz-Montes E., Yáñez-Fernández J., Castro-Muñoz R. (2021). Characterization of oligodextran produced by *Leuconostoc mesenteroides* SF3 and its effect on film-forming properties of chitosan. Mater. Today Commun..

[58] Muraro G.B., Carvalho-Estrada P.D., Pasetti M.H.D., Santos M.A., Nussio L.G. (2021). Bacterial dynamics of sugarcane silage in the tropics. Environ. Microbial..

[59] Koju R., Miao S., Liang B., Joshi D.R., Bai Y., Liu R., Qu J. (2020). Transcriptional and metabolic response against hydroxyethane-(1,1-bisphosphonic acid) on bacterial denitrification by a halophilic *Pannonibacter* sp. strain DN. Chemosphere.

[60] Liu Y., Pei T., Du J., Deng M.-R., Zhu H. (2021). *Inhella proteolytica* sp. nov. and *Inhella gelatinilytica* sp. nov., two novel species of the genus *Inhella* isolated from aquaculture water. Arch. Microbiol..

[61] Berrios L., Ely B. (2020). Plant growth enhancement is not a conserved feature in the *Caulobacter* genus. Plant Soil.

[62] Abdelazez A., Abdelmotaal H., Evivie S.E., Bikheet M., Sami R., Mohamed H., Meng X. (2022). Verification of *Lactobacillus brevis* tolerance to simulated gastric juice and the potential effects of postbiotic gamma-aminobutyric acid in streptozotocin-induced diabetic mice. Food Sci. Hum. Well..

[63] Mu L., Wang Q., Cao X., Zhang Z. (2021). Effects of fatty acid salts on fermentation characteristics, bacterial diversity and aerobic stability of mixed silage prepared with alfalfa, rice straw and wheat bran. J. Sci. Food Agric..

[64] Barrangou R., Yoon S.S., Breidt F., Fleming H.P., Klaenhammer T.R. (2002). Identification and characterization of *Leuconostoc fallax* strains isolated from an industrial sauerkraut fermentation. Appl. Environ. Microb..

[65] Zhang Q., Wu Z., Yu Z., Na R. (2018). Effect of lactic acid bacteria inoculants on fermentation characteristics and aerobic stability of three native grasses in Mongolian steppe. Grassl. Sci..

[66] Daniel J.L.P., Checolli M., Zwielehner J., Junges D., Fernandes J., Nussio L.G. (2015). The effects of *Lactobacillus kefiri* and *L. brevis* on the fermentation and aerobic stability of sugarcane silage. Anim. Feed Sci. Tech..

[67] Chiş M.S., Păucean A., Man S.M., Mureşan V., Socaci S.A., Pop A., Stan L., Rusu B., Muste S. (2020). Textural and sensory features changes of gluten free muffins based on rice sourdough fermented with *Lactobacillus spicheri* DSM 15429. Foods.

[68] Langille M.G.I., Zaneveld J., Caporaso J.G., McDonald D., Knights D., Reyes J.A., Clemente J.C., Burkepile D.E., Thurber R.L.V., Knight R. (2013). Predictive functional profiling of microbial communities using 16S rRNA marker gene sequences. Nat. Biotechnol..

