# Supplementary material for: Effects of Malic Acid and Sucrose on the Fermentation Parameters, CNCPS Nitrogen Fractions, and Bacterial Community of Moringa oleifera Leaves Silage
Source: Microorganisms. 2021 Oct 6;9(10):2102. doi: 10.3390/microorganisms9102102 (PMC8538485; doi:10.3390/microorganisms9102102)

Figure S1 The top 20 predicted functions of the bacterial communities analyzed via PICRUSt. CON, control group; MLA, 1% malic acid addition on the fresh weight (FW) basis; SUC, 1% sucrose addition on the FW basis; MIX, 1% malic acid and 1% sucrose addition on the FW basis.

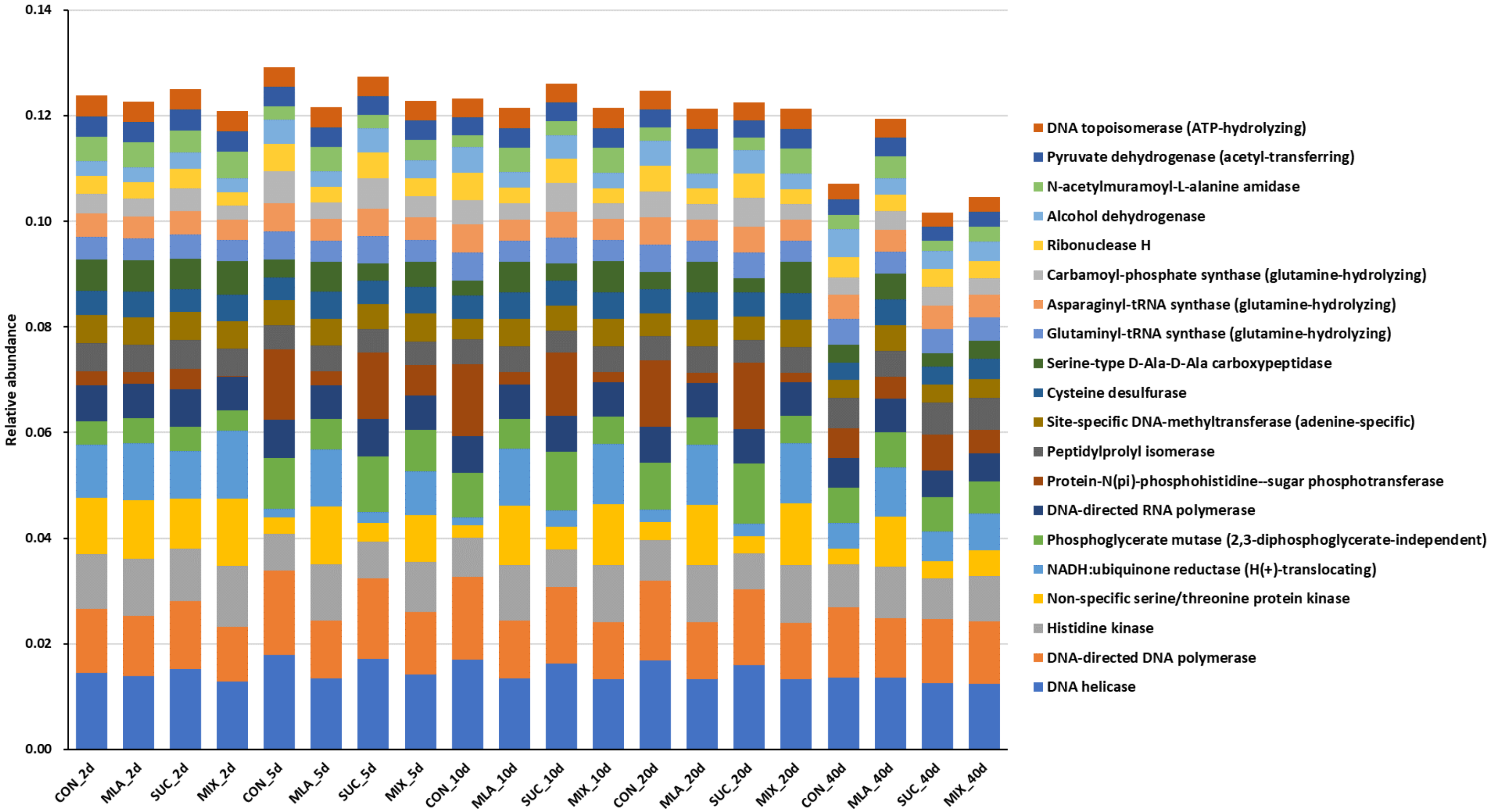

Figure S2 The top 20 predicted pathways of the bacterial communities analyzed via PICRUSt. CON, control group; MLA, 1% malic acid addition on the fresh weight (FW) basis; SUC, 1% sucrose addition on the FW basis; MIX, 1% malic acid and 1% sucrose addition on the FW basis.

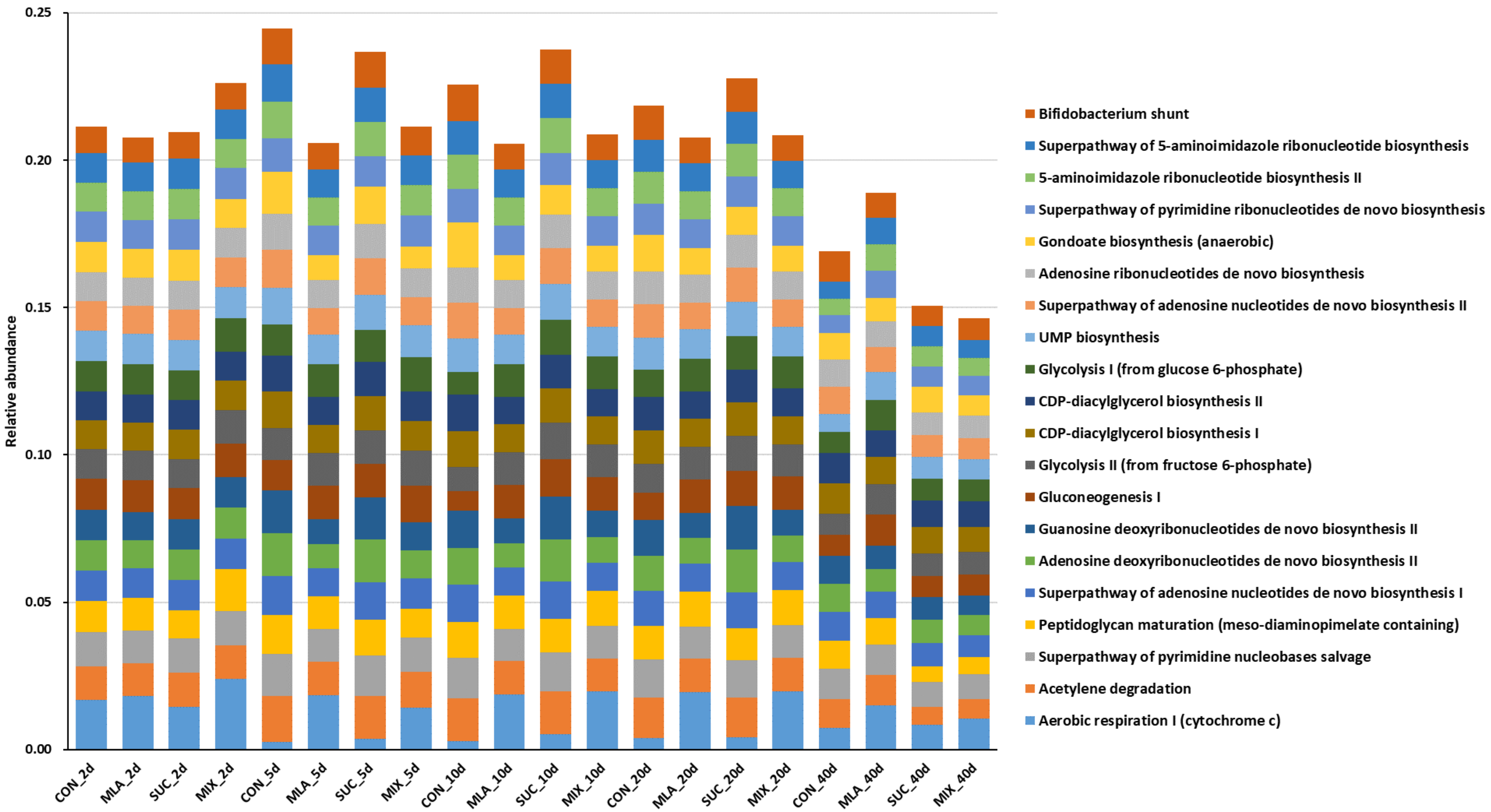

Supplement: Supplementary file 1 [file microorganisms-09-02102-s001.zip › Suppl_File_revised/Figure S1 and S2.pdf]
